# Reversible bone pain and symmetric bone scan uptake in a dialysis patient treated with cinacalcet: a case report

**DOI:** 10.1186/1752-1947-4-191

**Published:** 2010-06-24

**Authors:** Oliver Lenz, Rhea Sancassani, Carla Bottino, Alessia Fornoni

**Affiliations:** 1 Division of Nephrology and Hypertension, University of Miami Miller School of Medicine, Miami, Florida, USA; 2 Department of Medicine, Jackson Memorial Hospital, Miami FL, USA; 3 Universidad San Martin de Porres, Lima, Peru; 4 Diabetes Research Institute, University of Miami Miller School of Medicine, Miami, Florida, USA

## Abstract

**Introduction:**

The medical management of secondary hyperparathyroidism in patients with end-stage renal disease involves a combination of dietary restrictions, phosphate binders, active vitamin D analogs, and calcimimetics.

**Case presentation:**

We report the case of a 36-year-old Hispanic dialysis patient, originally from Cuba and now residing in the USA, who developed severe bone pain and muscle twitching after starting low dose cinacalcet, despite normal pre-dialysis ionized calcium and elevated parathyroid hormone. The clinical symptoms correlated with increased symmetrical uptake on bone scan that resolved rapidly upon discontinuation of cinacalcet.

**Conclusion:**

Cinacalcet may induce severe bone pain and a unique bone scan uptake pattern in hemodialysis patients.

## Introduction

The medical management of secondary hyperparathyroidism (SHPT) in patients with end-stage renal disease (ESRD) involves a combination of dietary restrictions, phosphate binders, active vitamin D analogs, and calcimimetics. Treatment is aimed at achieving the goals delineated in the Kidney Disease Outcomes Quality Initiative in order to avoid the adverse mineral metabolic abnormalities, including renal bone disease and cardiovascular mortality.

Cinacalcet (Sensipar^®^, Amgen, USA), a type II calcimimetic, allosterically increases the sensitivity of the calcium-sensing receptor, lowering the threshold for activation, and thereby decreasing secretion of parathyroid hormone (PTH) [1]. Studies have demonstrated its ability to lower PTH without significantly increasing serum calcium, phosphorus, or the Ca × P product [2]. Although symptomatic hypocalcemia may be a concern in pre-dialysis patients treated with cinacalcet [3], we only found one case report of cinacalcet-induced symptomatic hypocalcemia in a dialysis patient. This was accompanied by bone pain and thought to be a result of hungry bone syndrome caused by the too rapid correction of PTH [4], similar to what can be observed in a patient with severe SHPT following parathyroidectomy or kidney transplantation. In contrast, we report the case of a patient who developed severe bone pain and muscle twitching after starting low dose cinacalcet, despite normal pre-dialysis ionized calcium and elevated PTH.

## Case presentation

The patient is a 36-year-old Hispanic man, originally from Cuba and now residing in the USA, with hypertension and ESRD secondary to focal segmental glomerulosclerosis who had been undergoing hemodialysis for the past two years using a right radiocephalic fistula. He presented to a community hospital complaining of excruciating, constant, bilateral pain in both legs below the knees and the left forearm. He described the pain as dull and aching with periods of sharp non-radiating pain that required treatment with high dose narcotics. A ^99m^Tc bone scan performed on initial presentation showed bilateral symmetric uptake in the proximal tibia and distal femur, increased uptake in the metacarpophalangeal joints of the left hand, and bilateral radial and ulnar uptake. The axial skeleton and ribs were not involved. Although these findings were consistent with a metabolic rather than an inflammatory or infectious process and all blood cultures were negative, he received intravenous vancomycin and gentamycin for presumed osteomyelitis because he had undergone laparascopic cholecystectomy for acute cholecystitits and gram-negative sepsis four weeks prior to this admission. After 12 days of treatment our patient's pain had progressed in intensity and he transferred his care to our facility.

Our patient's past medical history was significant for focal segmental glomerulosclerosis leading to ESRD, hypertension, a seizure disorder, anemia of chronic disease, and SHPT. His medications included nifedipine, metoprolol, levetiracetam, cetirizine, calcium acetate, and cinacalcet; the latter had been started four weeks prior to the admission when our patient's intact PTH concentration in the serum was 750 pg/ml. The patient was now taking 30 mg daily.

At the time he presented to our institution our patient's pain continued. It was associated with fasciculations of the face and extremities, intermittent fevers, generalized weakness, and diarrhea. Physical examination showed a blood pressure of 170/95, heart rate of 105 beats per minute, respiratory rate of 18 per minute, temperature of 38.4°C, and 99% oxygen saturation on room air. Otherwise, his examination was only remarkable for muscle fasciculations in the face and upper extremities and a functioning right radiocephalic fistula.

Laboratory data revealed a white blood cell count of 5.8 × 10^3^/μL (6 × 10^9^/L) with normal differential, hemoglobin 11.6 g/dL (116 g/L), and platelets 267 × 10^3^/μL (267 × 10^9^/L). Serum chemistries were significant for blood urea nitrogen (BUN) 60 mg/dL (21.4 mmol/L), creatinine 11.1 mg/dL (846 μmol/L), calcium 8.7 mg/dL (2.17 mmol/L), ionized calcium 1.06 mEq/L (0.53 mmol/L), phosphorus 6.8 mg/dL (2.2 mmol/L), albumin 3.3 g/dL (33 g/L), alkaline phosphatase 465IU/L, PTH 659 pg/ml (659 ng/L). A Doppler ultrasound of the upper extremity did not reveal a deep venous thrombosis. X-rays of our patient's legs and left arm did not reveal any pathology.

**Figure 1:**
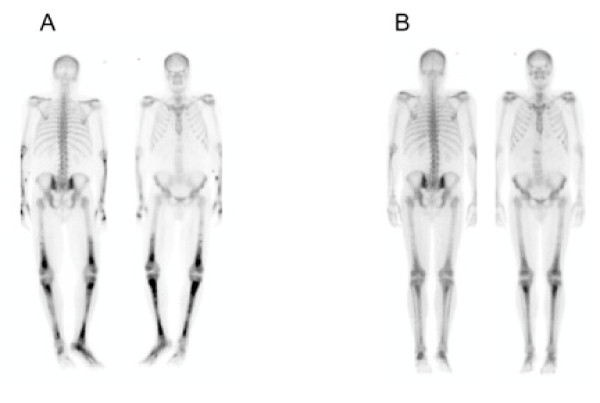
**Tc99 bone scan uptake during (A) and six weeks after discontinuation (B) of cinacalcet**.

Pain control was attempted with intravenous morphine but remained suboptimal. Given his intermittent fevers, blood cultures and stool studies were requested, and a bone scan was ordered to evaluate for osteomyelitis. All blood cultures remained negative, but stool studies were positive for *Clostridium difficile *toxin. Based on the clinical picture, a presumptive diagnosis of pseudomembranous colitis due to use of broad-spectrum antibiotics was made, and he was subsequently treated with oral metronidazole leading to resolution of his fevers. The ^99m^Tc bone-scan again showed an abnormal pattern of uptake involving symmetric bilateral upper and lower extremities with no involvement of the axial skeleton (Figure 1A). With the intent to perform a bone biopsy a computed tomography scan of our patient's lower extremities was performed, which did not reveal any focal findings. Similarly, magnetic resonance imaging of his lower extremities showed a preserved bony cortex with no focal findings.

Several metabolic conditions were considered to explain the ^99m^Tc uptake pattern [5]. However, the clinical presentation of our patient was inconsistent with any of the most plausible diagnoses, such as Paget's disease, rheumatoid arthritis, hypervitaminosis D, or osteomalacia. Our patient underwent hemodialysis three times weekly using a bicarbonate-based dialysate with a calcium concentration of 1.25 mmol/L. He achieved an adequate clearance, as measured by a Kt/V of 1.2 or greater, making uremic neuropathy an unlikely explanation, and his neurological examination did not suggest a neuropathic picture, which is why nerve conduction studies were not performed. Similarly, renal osteodystrophy appeared unlikely given the very symmetrical uptake. Given that calcium-sensing receptors have been reported to be present in bone, we hypothesized that cinacalcet may have caused increased osteoblastic activity, and consequently cinacalcet was stopped on day eight of hospitalization. Two days after discontinuation our patient's pain resolved and he was weaned off all analgesia. Muscle fasciculation also resolved in the absence of any change in ionized calcium, which had remained normal. Vitamin D analogues were prescribed for the management of his secondary hyperparathyroidism at discharge. A repeat bone scan six weeks later showed none of the abnormalities seen on the previous studies (Figure 1B). His PTH had decreased and the serum calcium stayed normal. At his six-week follow-up visit after discharge, he remained pain free.

## Conclusions

This case demonstrates that adverse effects from treatment with a calcium-receptor agonist should be considered in the differential diagnosis when evaluating a hemodialysis patient presenting with severe bone pain.

Calcium-sensing receptors (CaSR) can sense the extracellular calcium ion concentration and enable key tissues to maintain calcium homeostasis. Their expression in the proximal tubule, distal convoluted tubule, thick ascending limb of Henle, and chief cells of the parathyroid gland has been described and fully characterized. However, more recent data suggest novel localization of CaSRs on human arteries, breast cancer cells, as well as bones [6]. While it has been thought that a change in extracellular calcium concentration is necessary to activate CaSRs, the localization of CaSR on the membrane of the endoplasmic reticulum and the Golgi apparatus [7] suggests that CaSR may be involved in the regulation of intracellular calcium distribution independently of the extracellular calcium. A recent animal model with a constitutively active CaSR in osteoblasts suggests that CaSR activity may be independent of systemic changes in serum calcium or PTH concentrations [8]. Thus, it may be possible that our patient developed muscle fasciculations as a direct consequence of intracellular calcium redistribution despite a normal serum ionized calcium concentration. Similarly, the findings of symmetrical increase in osteoblastic activity by bone scan in the presence of normal ionized calcium may be explained by the presence of agonistic activity of cinacalcet on bone CaSR irrespective of the calcium concentration. In fact, it has been recently shown that CaSR may play a pivotal role in the control of both osteoclast and osteoblast differentiation as well as in the localization of hematopoietic progenitor stem cells to the bone endosteal surface. The evidence of cinacalcet induced increase in bone mineral density in long bones but not in the lumbar spine is consistent with the findings we have observed in our patient by bone scan [9]. It is possible that a specific pattern of CaSR expression in our patient has been responsible for the described findings. These could not be verified by polymerase chain reaction (PCR) on a bone biopsy specimen since our patient refused the procedure while hospitalized and there was no reason to perform a biopsy at a later point once he was completely asymptomatic. Our patient did not receive any active forms of vitamin D while taking cinacalcet. This is noteworthy given that almost all patients on maintenance hemodialysis receive active vitamin D sterols, and that hemodialysis patients who participated in past clinical trials with cinacalcet received concomitant treatment with vitamin D. It is conceivable that the lack of vitamin D therapy contributed to our patient's presentation. Unfortunately, we were unable to follow the patient long-term and hence we do not know if the patient was re-challenged with cinacalcet after starting an active vitamin D analog.

This case does not prove definitive evidence for a causal relationship between our patient's bone pain, abnormal bone scan, and cinacalcet use. Nevertheless, we believe that the clinician faced with a similar constellation should at least consider the possibility that cinacalcet may induce severe bone pain and a unique bone scan uptake pattern in hemodialysis patients.

## Consent

Written informed consent could not be obtained despite all reasonable attempts. Every effort has been made to protect the identity of the patient and there is no reason to think that the patient or their family would object to this publication.

## Competing interests

The authors declare that they have no competing interests.

## Authors' contributions

RS and CB compiled and analyzed the clinical data. RS, CB, OL, and AF were major contributors in writing the manuscript. All authors read and approved the final manuscript.

## Editor's note

This manuscript was submitted prior to our change of policy on consent.

## References

[B1] Valle C Rodriguez M Santamaria R Almaden Y Rodriguez ME Canadillas S Martin-Malo A Aljama P Cinacalcet reduces the set point of the PTH-calcium curve J Am Soc Nephrol 2008 19 2430 2436 10.1681/ASN.2007121320 18632847 PMC2588095

[B2] Fishbane S Shapiro WB Corry DB Vicks SL Roppolo M Rappaport K Ling X Goodman WG Turner S Charytan C Cinacalcet HCl and concurrent low-dose vitamin D improves treatment of secondary hyperparathyroidism in dialysis patients compared with vitamin D alone: the ACHIEVE study results Clin J Am Soc Nephrol 2008 3 1718 1725 10.2215/CJN.01040308 18945995 PMC2572296

[B3] Chonchol M Locatelli F Abboud HE Charytan C de Francisco AL Jolly S Kaplan M Roger SD Sarkar S Albizem MB Mix TC Kubo Y Block GA A randomized, double-blind, placebo-controlled study to assess the efficacy and safety of cinacalcet HCl in participants with CKD not receiving dialysis Am J Kidney Dis 2009 53 197 207 10.1053/j.ajkd.2008.09.021 19110359

[B4] Lazar ES Stankus N Cinacalcet-induced hungry bone syndrome Semin Dial 2007 20 83 85 10.1111/j.1525-139X.2007.00248.x 17244128

[B5] Buckley O O'Keeffe S Geoghegan T Lyburn ID Munk PL Worsley D Torreggiani WC 99 mTc bone scintigraphy superscans: a review Nucl Med Commun 2007 28 521 527 10.1097/MNM.0b013e3281744440 17538392

[B6] Yamaguchi T The calcium-sensing receptor in bone J Bone Miner Metab 2008 26 301 311 10.1007/s00774-008-0843-7 18600395

[B7] Tu CL Chang W Bikle DD The role of the calcium sensing receptor in regulating intracellular calcium handling in human epidermal keratinocytes J Invest Dermatol 2007 127 1074 1083 10.1038/sj.jid.5700633 17124506

[B8] Dvorak MM Chen TH Orwoll B Garvey C Chang W Bikle DD Shoback DM Constitutive activity of the osteoblast Ca2+-sensing receptor promotes loss of cancellous bone Endocrinology 2007 148 3156 3163 10.1210/en.2007-0147 17412806

[B9] Lien YH Silva AL Whittman D Effects of cinacalcet on bone mineral density in patients with secondary hyperparathyroidism Nephrol Dial Transplant 2005 20 1232 1237 10.1093/ndt/gfh829 15840675

